# Significance of Serum p53 Antibody as a Tumor Marker in Colorectal Cancer

**DOI:** 10.1155/2019/2721876

**Published:** 2019-12-14

**Authors:** Rina Takahashi, Kazuhiro Sakamoto, Kiichi Sugimoto, Shunsuke Motegi, Ryoichi Tsukamoto, Ryosuke Ichikawa, Yu Okazawa, Jun Aoki, Shun Ishiyama, Makoto Takahashi, Yutaka Kojima, Atsushi Okuzawa, Yuichi Tomiki, Joe Matsuoka

**Affiliations:** ^1^Department of Coloproctological Surgery, Juntendo University Faculty of Medicine, 2-1-1 Hongo, Bunkyo-ku, Tokyo 113-8421, Japan; ^2^Innovative Medical Technology Research & Development Center, Juntendo University, 2-1-1 Hongo, Bunkyo-ku, Tokyo 113-8421, Japan

## Abstract

**Objective:**

We examined serum anti-p53 antibodies (S-p53Ab) in colorectal cancer. Specifically, we retrospectively investigated the use of S-p53Ab as a prognostic marker after surgery for colorectal cancer.

**Materials and Methods:**

The levels of S-p53Ab, carcinoembryonic antigen (CEA), and carbohydrate antigen 19-9 (CA19-9) were measured in 160 colorectal cancer patients who underwent surgical treatment. The rate of postoperative change (RPC) in the S-p53Ab titer was calculated as [subsequent antibody titer-lowest titer]/lowest titer.

**Results:**

A relationship between recurrence and RPC in the S-p53Ab titer was not observed in patients who tested negative for S-p53Ab preoperatively. In addition, no patients, who tested negative for S-p53Ab preoperatively, tested positive for S-p53Ab at the follow-up after surgery. Of the patients who tested positive for S-p53Ab preoperatively, those recurrences had a significantly higher RPC compared with those who did not (*p* < 0.001).

**Conclusions:**

Although S-p53Ab is not a significant tumor marker in patients who test negative preoperatively, increases in the S-p53Ab titer should be continuously monitored and measured in patients who are positive for this antibody preoperatively, regardless of whether they later test negative.

## 1. Introduction

Tumor markers are widely used in screening, diagnosis, monitoring, and prognosis of various cancers. Currently, carcinoembryonic antigen (CEA) and carbohydrate antigen 19-9 (CA19-9) are used as markers for monitoring colorectal cancer. While conventional tumor markers are produced by cancer cells, anti-p53 antibodies are autoantibodies against proteins originating from cancer cells and have recently garnered attention [1, 2].

The study on dysfunction of the oncogene in digestive organ cancer is conducted widely, and the thing that the mutation of p53 variation is common is reported. In addition, the mutation of p53 and perturbation of its function are common in human malignancies [3, 4]. The half-life of the mutant p53 protein in clinical samples has been reported to be several hours, whereas that of the wild-type p53 protein is only 20 min. The accumulation of p53 gene proteins in the nuclei of malignant cells induces the production of serum anti-p53 antibodies (S-p53Ab) [3, 4]. Previous studies reported that S-p53Ab is a useful diagnostic marker for early cancer because microvolumes of mutant p53 protein are detectable [1–4], but there are only a few reports on S-p53Ab as a predictor of long-term outcomes after surgery [5]. Indeed, some patients remain positive for S-p53Ab after surgical resection without recurrence for several years. However, the meaning of the postoperative change in the S-p53Ab titer is unknown.

Therefore, in this study, we examined S-p53Ab as a prognostic marker to predict the long-term outcome after surgery.

## 2. Materials and Methods

### 2.1. Patients

A total of 160 patients with primary colorectal adenocarcinoma who underwent surgical treatment at our hospital between September 2008 and September 2011 were enrolled in this study. Patients with multiple primary colorectal cancers and double cancers were excluded. All clinical data relevant to the patients were obtained from medical records. Staging was performed using the TNM classification (8^th^ ed. [6]). Although patients with stage I-III cancer underwent curative resection, those with stage IV cancer did not.

### 2.2. Methods

The tumor markers were measured preoperatively and every 3 months postoperatively. Metastasis and recurrence were assessed via CT scan every 6 months in accordance with the guidelines of the Japanese Society for Cancer of the Colon and Rectum [7].

### 2.3. Measurement of S-p53Ab Levels and Assays for CEA, CA19-9

We followed the methods of Ochiai et al. [8]. The levels of S-p53Ab were assessed using an ELISA Kit MESACUP anti-p53 Test (MEDICAL & BIOLOGICAL LABORATORIES, Nagoya, Japan). Briefly, samples were added to the wells of a microtiter plate coated with either wild-type human p53 or control protein and incubated for 1 h. Peroxidase-conjugated goat anti-human immunoglobulin G-binding S-p53Ab was then added and incubated for another 1 h followed by the addition of substrate solution and incubation for 30 min. A calibration curve was constructed from the specific signals of standards and from the levels of antibodies indicated on the vials containing the standards. The cutoff value was 1.3 U/ml.

CEA concentrations were measured using a CEA-II EIA kit (Roche Diagnostics, Tokyo, Japan). The cutoff value for serum CEA was 5.0 ng/ml. CA19-9 concentrations were measured using a Roche Diagnostics kit (Tokyo, Japan). The cutoff value for serum CA19-9 was 37 U/ml.

### 2.4. Immunohistochemical Expression of p53

**Figure 1:**
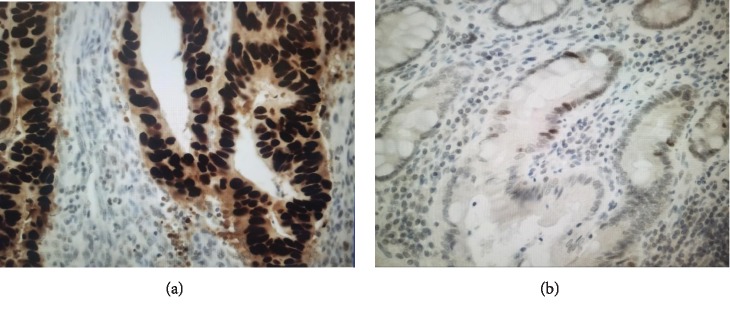
Immunohistochemical expression of p53. A tumor containing more than 10% brown-stained cell nuclei was considered positive. (a) Positive case: clear positive staining in 94.7% (×400). (b) Negative case: fewer positive cells and weak staining (×200).

The expression of p53 was immunohistochemically examined in all patients who were positive for S-p53Ab preoperatively and in representative patients (20 patients) who were negative for S-p53Ab preoperatively. This test was performed as described previously [8, 9]. Briefly, immunohistochemical staining was performed using mouse anti-human p53 monoclonal antibody (DO-7 M7001, Dako, Glostrup, Denmark) on 4 *μ*m thick serial sections of formalin-fixed, paraffin-embedded colorectal cancer. A tumor in which more than 10% of the 1200~1800 counted cell nuclei were stained was considered to be positive (at ×400). The results were evaluated by two independent researchers (Figure 1).

### 2.5. Statistical Analysis

Statistical analysis was performed using JMP (Ver. 12). Fisher's exact test and Student's *t*-test were used to evaluate differences between the variables, and *p* values < 0.05 were considered significant.

The obtained data were used to calculate Kaplan-Meier estimates with metastasis or recurrence as an event, and the proportional hazards assumption was verified. Positivity for S-p53Ab served as a time-dependent covariate in a Cox model, and a final model was determined using variable selection.

We evaluated the rate of postoperative change (RPC) in the S-p53Ab titer as follows. First, the lowest antibody titer was designated as “lowest titer,” and the antibody titer after the lowest titer was designated as “subsequent antibody titer.” The RPC in the S-p53Ab titer was calculated as [subsequent antibody titer-lowest titer]/lowest titer.

## 3. Results

### 3.1. Patient Characteristics

There were 45 patients with stage I disease, 48 with stage II disease, 59 with stage III disease, and 8 with stage IV disease. The subjects included 93 men and 67 women with a median age of 65 years (range, 35-90 years). The postoperative follow-up was 4 years and 7 months (range, 10 months-6 years).

### 3.2. Preoperative S-p53Ab Titers and Clinicopathologic Factors

The relationship between S-p53Ab titers and clinicopathologic factors was examined in 160 patients who underwent surgical resection. Patients who tested positive for S-p53Ab were more likely to have cancer in the rectum rather than the colon. The S-p53Ab titer was not related to age, gender, depth of tumor, status of lymph node, status of lymphatic invasion, status of vessel invasion, stage, increases in CEA and CA19-9 levels, or recurrence. In 152 patients (excluding those with stage IV cancer), a relationship between preoperative S-p53Ab levels and recurrence was not observed (Table 1).

### 3.3. Immunohistochemical Expression of p53

The expression of p53 was immunohistochemically examined in all of the patients (37 patients) who were positive for S-p53Ab preoperatively and in representative patients (20 patients) who were negative for S-p53Ab preoperatively. The relationship between S-p53Ab and p53 expression in the nucleus of cancer cells is shown in Table 2. S-p53Ab preoperatively was positive in 32 patients (84.2%) of immunostaining-positive cases and negative in 14 patients (73.7%) of immunostaining-negative cases (*p* < 0.001).

### 3.4. Related Factors for Recurrence by a Cox Model

When analyzing factors that are related to recurrence, positivity for S-p53Ab served as a time-dependent covariate in a Cox model. This yielded a model with age, gender, location of tumor, depth of tumor, status of lymph node, status of lymphatic invasion, status of vessel invasion, stage, and increases in CEA and CA19-9 levels as explanatory variables (Table 3).

### 3.5. Changes in S-p53Ab Levels Based on Preoperative Values

**Figure 2:**
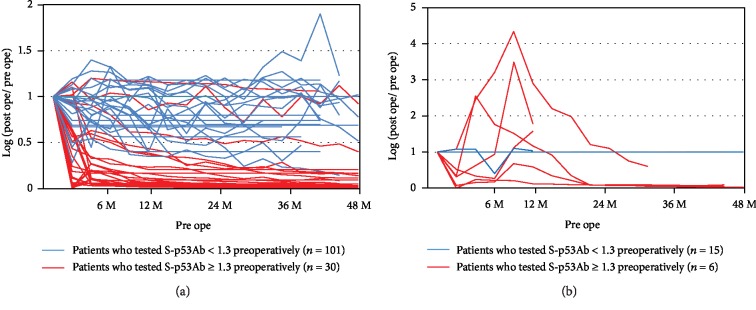
Changes in S-p53Ab levels based on preoperative values: (a) patients with no recurrence; (b) patients with recurrence. In most patients who tested positive for S-p53Ab preoperatively and did not experience recurrence, the levels of S-p53Ab decreased. In most patients who tested positive for S-p53Ab preoperatively and had recurrence, there was a temporary increase in the S-p53Ab levels.

Of the 152 patients who were assessed (excluding those with stage IV cancer), 21 (13.8%) had recurrence. The postoperative values based on preoperative values from the S-p53Ab titer were examined. In most patients who tested positive for S-p53Ab preoperatively and did not experience recurrence, the levels of S-p53Ab decreased (Figure 2(a)). In most patients who tested positive for S-p53Ab preoperatively and had recurrence, there was a temporary increase in the S-p53Ab levels (Figure 2(b)). Furthermore, none of the patients who were negative for S-p53Ab preoperatively became positive postoperatively.

### 3.6. Postoperative Changes over Time and Recurrence

**Figure 3:**
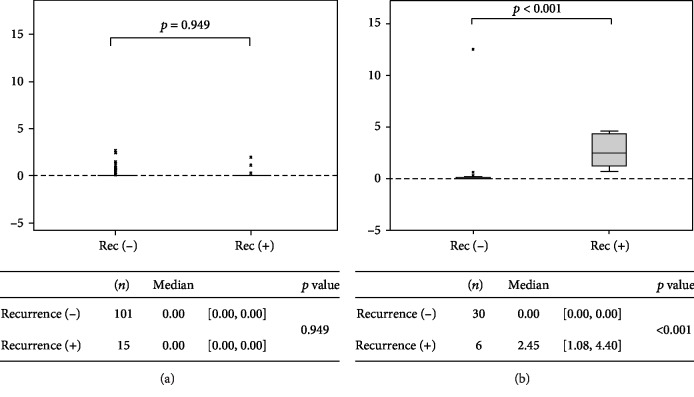
Relationship between the rate of increase and recurrence: (a) preoperative S − p53Ab levels < 1.3; (b) preoperative S − p53Ab levels ≥ 1.3. Of the patients who tested positive for S-p53Ab preoperatively, those who had metastasis or recurrence had significantly higher S-p53Ab titers compared with those who did not experience recurrence.

Of the 116 patients who tested negative for S-p53Ab preoperatively, 15 (12.9%) had recurrence. In patients who tested negative for S-p53Ab preoperatively and had recurrence, the RPC in the S-p53Ab titer was 0.00 (IQR: 0.00-0.00) (Figure 3(a)). In patients who tested negative for S-p53Ab preoperatively and did not experience recurrence, the RPC in the S-p53Ab titer was 0.00 (IQR: 0.00-0.00) (Figure 3(a)). Therefore, a relationship between recurrence and RPC in the S-p53Ab titer was not observed (*p* = 0.949) (Figure 3(a)).

Of the 36 patients who tested positive for S-p53Ab preoperatively, 6 (16.7%) had recurrence. In patients who tested positive for S-p53Ab preoperatively and had recurrence, the RPC in the S-p53Ab titer was 2.45 (IQR: 1.08-4.40) (Figure 3(b)). In patients who tested positive for S-p53Ab preoperatively and did not experience recurrence, the RPC in the S-p53Ab titer was 0.00 (IQR: 0.00-0.00) (Figure 3(b)). Of the patients who tested positive for S-p53Ab preoperatively, those with recurrence had significantly higher S-p53Ab titers compared with those who did not (*p* < 0.001) (Figure 3(b)).

### 3.7. Marker for Recurrence

**Figure 4:**
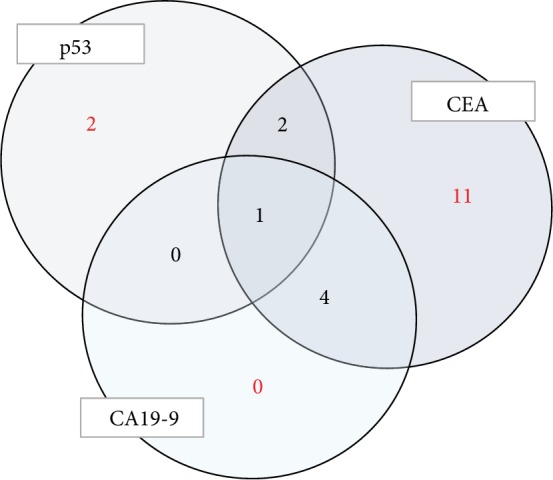
Number of patients with elevated tumor markers during recurrence. The S-p53Ab level was elevated in only 5 patients. In the 3 patients with recurrence, both S-p53Ab and CEA levels were elevated.

In 21 patients with recurrence, the levels of some markers were elevated. The S-p53Ab level was elevated in only 5 patients, the CEA level was elevated in 18, and the CA19-9 level was elevated in 5. In the 3 patients with recurrence, both S-p53Ab and CEA levels were elevated. And all three levels were elevated in only 1 patient (Figure 4).

## 4. Discussion

We observed a higher frequency of S-p53Ab positivity in rectal cancer than in colon cancer. However, we found that there was no correlation between the positivity of S-p53Ab and clinicopathological factors, which is similar to other reports [5, 10].

And we examined the immunohistochemical expression of p53 to assess the detectability of S-p53Ab. Preoperatively, S-p53Ab was positive in 84.2% of immunostaining-positive patients. S-p53Ab may indicate the presence of p53 gene mutation. On the other hand, 15.8% of immunostaining-positive patients were negative for S-p53Ab preoperatively. The sensitivity and specificity differed in previous reports, and the false-negative rate widely differs among studies [8, 9, 11]. As there are many mutations of the p53 gene, the immunoresponse varies according to the difference in subtype and different mutated p53 proteins may not appear in the serum [12, 13]. As another cause, the cutoff value of S-p53Ab may be related.

Some studies have suggested that S-p53Ab positivity is associated with poor prognosis [11, 14, 15]; however, others have indicated that S-p53Ab is not a good prognostic marker [16]. In patients with colorectal cancer, a correlation between S-p53Ab and clinicopathological features or colorectal cancer prognosis has yet to be clarified. In this study, factors for colorectal cancer metastasis and recurrence were not associated with the preoperative values of S-p53Ab but with time-dependent changes in S-p53Ab level.

This study examined RPC in the S-p53Ab titer, regardless of whether the patients later tested negative for this antibody. The lowest titer at follow-up was used to calculate the RPC in the S-p53Ab titer, and the relationship between this RPC in the S-p53Ab titer and recurrence was examined. Importantly, we found that none of the patients who were negative for S-p53Ab preoperatively became positive postoperatively.

This study focused on RPC in the S-p53Ab titer in patients who tested positive for this antibody preoperatively and showed that S-p53Ab can be used as a prognostic marker of poor outcome in these patients. RPC in the S-p53Ab titer should be closely monitored in patients who test positive for this antibody preoperatively. If patients that are positive for S-p53Ab preoperatively show an increase in S-p53Ab levels, they should be examined for recurrence.

There are some limitations to this study. First, the sample size is small since this study examined patients from only a single facility. Second, there was no validation study.

Based on the findings of this study, RPC in the S-p53Ab titer is a valuable marker for recurrence in patients who test positive for S-p53Ab preoperatively in colorectal cancer. In future studies, it is necessary to examine a larger number of patients who test positive for S-p53Ab preoperatively.

## 5. Conclusion

Although S-p53Ab is not a significant tumor marker in patients who test negative preoperatively, increases in the S-p53Ab titer should be continuously monitored and measured in patients who are positive for this antibody preoperatively, regardless of whether they later test negative.

## Figures and Tables

**Table 1:** Patient characteristics.

	Preoperative S-p53Ab				*p* value
	<1.3 (*n* = 123)		≥1.3 (*n* = 37)		
	*n*	%	*n*	%	
Age (years)	64 ± 10.8		62.8 ± 10.5		0.592
Gender					0.349
Male	74	60.2	19	51.4	
Female	49	39.8	18	48.6	
Location of tumor					0.022
Colon	80	65.0	16	43.2	
Rectum	43	35.0	21	56.8	
Depth of tumor					0.185
T1/T2	52	42.3	11	29.7	
T3/T4	71	57.7	26	70.3	
Status of lymph node					0.184
Positive	76	61.8	18	48.6	
Negative	47	38.2	19	51.4	
Status of lymphatic invasion					0.839
Positive	87	70.7	27	73.0	
Negative	36	29.3	10	27.0	
Status of vessel invasion					0.443
Positive	77	62.6	20	54.1	
Negative	46	37.4	17	45.9	
Stage					0.324
1	35	28.5	10	27.0	
2	41	33.3	7	18.9	
3	40	32.5	19	51.4	
4	7	5.7	1	2.7	
CEA					0.176
Positive	43	35.0	18	48.6	
Negative	80	65.0	19	51.4	
CA19-9					1.000
Positive	9	7.3	2	5.4	
Negative	114	92.7	35	94.6	
Recurrence (excluding stage 4)					0.275
Positive	15	12.9	6	16.7	
Negative	101	87.1	30	83.3	

**Table 2:** Immunohistochemical expression of p53.

	S-p53Ab		*p* value
	Positive (*n* = 37)	Negative (*n* = 20)	
Immunostaining of p53			
Positive (*n* = 38)	32 (84.2%)	6 (15.8%)	
Negative (*n* = 19)	5 (26.3%)	14 (73.7%)	<0.001

**Table 3:** Factors for metastasis or recurrence by a Cox model.

Clinicopathological factor	Degrees of freedom	Wald *χ*^2^ test	*p* value
S-p53Ab (time-dependent covariation)	1	6.9362	0.001
Age	1	0.1605	0.39
Gender	1	0.2787	0.598
Location of tumor	1	0.9884	0.561
Depth of tumor	1	5.6219	0.018
Status of lymph node	1	36.925	<0.001
Status of lymphatic invasion	1	1.5071	0.6806
Status of vessel invasion	1	16.8252	<0.001
Stage	1	15.3669	0.002
Increases in CEA levels	1	5.0372	0.024
Increases in CA19-9 levels	1	0.2405	0.064

## Data Availability

The data used to support the findings of this study are available from the corresponding author upon request.
